# Transformers meets neoantigen detection: a systematic literature review

**DOI:** 10.1515/jib-2023-0043

**Published:** 2024-07-04

**Authors:** Vicente Machaca, Valeria Goyzueta, María Graciel Cruz, Erika Sejje, Luz Marina Pilco, Julio López, Yván Túpac

**Affiliations:** 27840 Universidad La Salle, Arequipa, Perú; 187038 Universidad Católica San Pablo, Arequipa, Perú; Universidad Nacional de San Agustín, Arequipa, Perú; Fuerza Aérea del Perú, Arequipa, Perú

**Keywords:** deep learning, neoantigen, review, transformers, BERT, cancer

## Abstract

Cancer immunology offers a new alternative to traditional cancer treatments, such as radiotherapy and chemotherapy. One notable alternative is the development of personalized vaccines based on cancer neoantigens. Moreover, Transformers are considered a revolutionary development in artificial intelligence with a significant impact on natural language processing (NLP) tasks and have been utilized in proteomics studies in recent years. In this context, we conducted a systematic literature review to investigate how Transformers are applied in each stage of the neoantigen detection process. Additionally, we mapped current pipelines and examined the results of clinical trials involving cancer vaccines.

## 1 Introduction

Cancer represents the most significant global health challenge [1]. Furthermore, according to the Cancer Research Institute of the United Kingdom, more than 18 million new cases and 10 million deaths were recorded in 2020 [1]. Furthermore, it is predicted that there will be 28 million new cases annually by around 2040 if the incidence remains stable, and population growth and aging continue according to recent trends [2]. This represents a 54.9 % increase from 2020, with the increase expected to be higher in men (60.6 %) than in women (48.8 %).

In this context, it is well known that traditional methods based on surgery, radiotherapy, and chemotherapy have low efficacy and adverse side effects [3]. Thus, the development of cancer immunotherapy has emerged, aiming to stimulate the immune system of the patient [4]. There are treatments like personalized vaccines, adoptive T-cell therapies, and immune checkpoint inhibitors. Among these, neoantigen-based vaccines have shown great potential by enhancing T-cell responses and are considered the most likely to succeed [4]. Additionally, neoantigens are used in immune checkpoint blockade therapy. Neoantigens are considered predictive biomarkers and targets for synergistic treatment in cancer immunotherapy [5].

**Figure 1:**
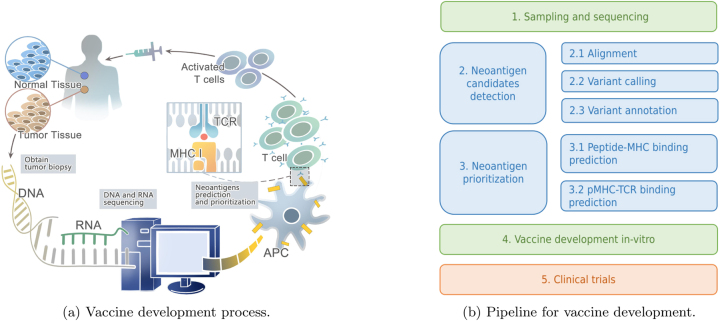
Development framework for the development of personalized cancer vaccines based on neoantigens. (a) Provides a general overview of each stage [9]. (b) A general overview of each phase with an emphasis on in-silico development.

The development of personalized cancer vaccines is a lengthy process dependent on the accurate detection of neoantigens (see Figure 1). These neoantigens are peptides found exclusively in cancer cells [6]. The goal of a personalized vaccine-based treatment is to train the lymphocytes (T-cells) of the patient to recognize these neoantigens and activate the immune system [3, 7]. The process is summarized in Figure 1b and consists of the following steps:

1. Get samples of cancerous and healthy tissues. Both tissues are then sequenced to obtain DNA and/or RNA. Some approaches include immunopeptidome information from Mass Spectrometry (MS).
2. In the in-silico stage, sequence alignment is performed, a variant calling process is developed to detect variations and/or mutations, and these variants are annotated (possible neoantigen detection). Several tools with good performance are available for this stage.
3. Neoantigens are prioritized in this in-silico stage. This step is crucial and has received significant research attention in recent years due to its complexity and the low effectiveness of current approaches. Here, candidate neoantigens (peptides) from the previous stage are assessed for their affinity with the Major Histocompatibility Complex (MHC), known as pMHC binding. Then, the affinity of pMHC to bind with the T-cell Receptor (TCR) is evaluated. At the end of this stage, neoantigens are obtained.
4. In the *in vitro* stage, the T-cells of the patient are induced in the laboratory to recognize the neoantigens. Vaccines are developed at this point. Typically, this stage is carried out by biotechnologists and biologists.
5. Finally, the oncologist conducts a clinical evaluation of the vaccine.

The *in-silico* detection of neoantigens is based on the second and third stages depicted in Figure 1b. In this context, due to the complexity of the process and the variety of methods available, software tools and pipelines have been developed to streamline the use of these tools. Moreover, Transformers has marked the beginning of a new era in artificial intelligence, showcasing notable achievements in a range of Natural Language Processing tasks (NLP) tasks [8]. These models have also found application in neoantigen detection, particularly in the third stage of Figure 1b. BERT models and deep learning networks with attentions mechanisms have been proposed for peptide-MHC and pMHC-TCR binding prediction.

Despite various efforts in the development of neoantigen detection methods, less than 5 % of detected neoantigens activate the immune system, as reported by several studies [7, 10], [11], [12], [13]. The reasons are related to: the no integration of multiple data sources like DNA-seq, RNA-seq, and Mass Spectrometry (MS) [14]. Use of low-performance tools for peptide-MHC binding prediction like MHCFlurry [15] and NetMHCpan4.1 [16]. Neglecting the prediction of pMHC-TCR binding [17]. Overlooking information from alternative splicing events, structural DNA variants, and gene fusion mutations, this information is closely related to various types of cancer [18].

In this work, we conducted a systematic literature review of the application of Transformers in neoantigen detection. This work represents an extension of a prior review [19] focused on pMHC binding prediction. Our primary focus is on neoantigen prioritization, as this area has seen a substantial amount of research utilizing Transformers. Additionally, we integrated pipeline analyses and clinical trial studies to gain insights into the latest findings regarding the application of neoantigen detection in personalized cancer vaccines. Our review distinguishes itself from others by taking a computational perspective and exclusively examining methods that leverage Transformers. Furthermore, our contributions encompass: (1) an updated review of methods for detecting cancer neoantigens using exclusively Transformers. (2) The inclusion of clinical trials to show the efficacy of these methods in the development of cancer vaccines.

## 2 Methodology

In order to review Transformer methods used in neoantigen detection, we performed a Systematic Literature Review (SLR). The search strings used are shown in Table 1.

**Table 1:** Search string used in the SLR for each phase of neoantigen detection.

Category	Search string
Neoantigen prioritization	“(mhc OR hla) AND (peptide OR epitope OR antigen) AND (specificity OR immunogenicity OR binding OR affinity OR predict* OR detection OR presentation OR classification) AND (transformer* OR bert* OR attention OR ‘transfer learning’ OR method* OR predict*)”, “(tcr OR ’t cell’ OR t-cell) AND (mhc OR peptide OR epitope OR antigen) AND (specificity OR immunogenicity OR binding OR affinity OR predict* OR detection OR presentation OR classification) AND (transformer* OR bert* OR attention OR ’transfer learning’ OR method* OR predict*)”
Pipelines	“(pipeline OR toolkit) AND (tcr OR t cell OR t-cell OR mhc OR hla OR peptide OR epitope OR antigen* OR neoantigen*) (pipeline OR tool* OR workflow OR application OR web*) AND (peptide OR epitope OR antigen* OR neoantigen* OR neoepito*) AND (immunotherapy OR detection OR identify* OR predict* OR presentation*)”
Clinical trials	“neoantigen OR neoepitope OR denditric cell) AND (vaccines OR immunology”

We proposed the following research questions: **Q1**. How Transformers are applied in neoantigen detection? **Q2**. What problems and limitations Transformer face in neoantigen detection? **Q3**. What future works arise from Transformers in neoantigen detection? **Q4**. What pipelines are used in neoantigen detection? **Q5**. How clinical trials are used in neoantigen detection?

Based on the search strings and considering only works since 2018, the titles of the articles were analyzed, obtaining 151 articles. Then, a subset was selected based on the inclusion criteria: articles with ERA category (A or B) or articles from Q1/Q2 journals. At the end of this stage, 79 articles were obtained.

## 3 Transformers

The concept of the attention mechanism was initially introduced by Bahdanau in 2014 [20] to address the limitations associated with fixed-length encoding vectors. This novel approach yielded comparable state-of-the-art results for English-to-French translation. Subsequently, the attention mechanism found application in natural language inference [21], leading to the proposal of a structured attention network [22]. However, it is worth noting that these attention modules were typically used in conjunction with recurrent networks. A significant shift occurred in 2017 with the publication of the groundbreaking paper “Attention Is All You Need” by Vaswani et al. [23], which introduced a novel network architecture known as the Transformer. This architecture relied exclusively on attention mechanisms and represented a fundamental departure from traditional approaches. In 2018, the bidirectional transformer model Bidirectional Encoder Representations from Transformers (BERT) was introduced by Devlin et al. [24]. It has since become one of the most widely recognized and influential transformer models in the field. Transformer relies on the concept of “self-attention”. It refers to how much attention a word attend to the other words. For instance, in the following sentence: “The animal didn’t cross the street because it was too tired”, self-attention allows to associate “it” with “animal” [25].

In this context the main block is the self-attention sa[⋅], which takes *N* inputs *x*_*n*_, each of dimention *D* × 1, and returns *N* outputs vectors of the same size. In NLP, each input *x*_*n*_ is a word; meanwhile in protein sequences, represent an amino acid. Then, a set of values are computed by *v*_*n*_ = *β*_*v*_ + Ω_*v*_ *x*_*n*_, where *β*_*v*_ and Ω_*v*_ are the biases and weights respectively. So, the self-attention block is compute by Equation (1). The weight *a*[*x*_*m*_, *x*_*m*_] is the attention that output *x*_*n*_ pays to *x*_*m*_.

(1) $$\text{sa}\left[x_{n}\right]=\overset{N}{\underset{m=1}{\sum }}a\left[x_{m},x_{n}\right]v_{m}$$

**Figure 2:**
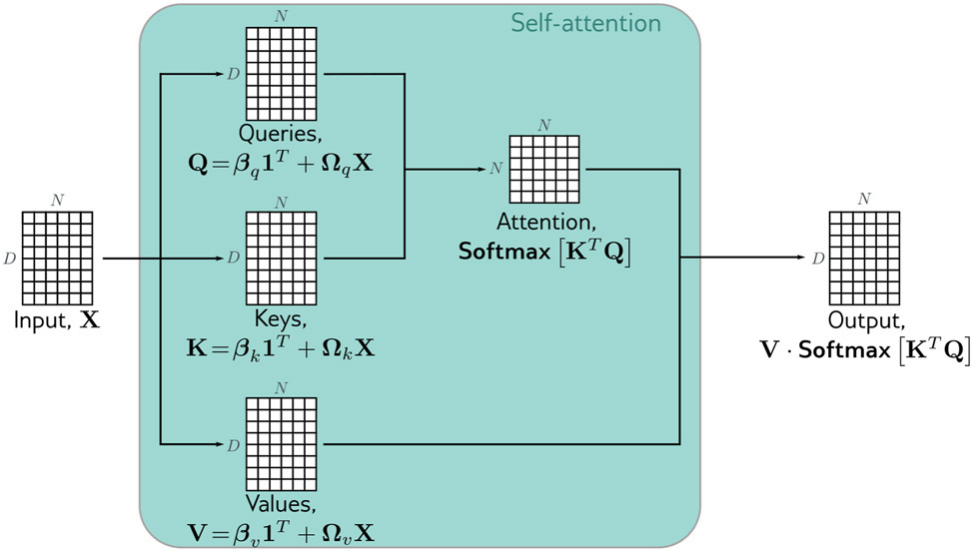
How to compute attention weights. Source: [25].

To compute attention, we apply these linear transformations: *q*_*n*_ = *β*_*q*_ + Ω_*q*_ *x*_*n*_, and *k*_*n*_ = *β*_*k*_ + Ω_*k*_ *x*_*n*_; where *q*_*n*_ and *k*_*k*_ are referred to as queries and keys, respectively. Then, the scaled dot-product attention is show in Equation (2). In Figure 2, we represent this dot-product attention method. Moreover, the self-attention in a matrix form is shown in Equation (3); however, the product can have large values, so this equation is scaled in Equation (4).

(2) $$a\left[x_{m},x_{n}\right]=\text{softmax}\left[k^{T}\cdot q_{n}\right]$$

(3) $$\text{Sa}\left[X\right]=V\cdot \text{softmax}\left[k^{T}\cdot q_{n}\right]$$

(4) $$\text{Sa}\left[X\right]=V\cdot \text{softmax}\left[\frac{k^{T}\cdot q_{n}}{\sqrt{D_{q}}}\right]$$

Moreover, multiple heads seem to be necessary to make the Transformer work well. So we concatenate several head attentions like Equation (5).

(5) $$\text{MhSa}\left[X\right]=\Omega_{c}\left[{\text{Sa}}_{1}\left[X\right];{\text{Sa}}_{2}\left[X\right];\ldots ;{\text{Sa}}_{H}\left[X\right];\right]$$

Moreover, another crucial aspect of Transformers involves positional encoding. In this process, we incorporate information about the position of elements within the sequence. A common strategy for positional encoding utilizes sine and cosine functions with varying frequencies. With this method, each dimension of the positional encoding corresponds to a sine or cosine function, contributing to a comprehensive representation of sequence positions [25, 26].

Furthermore, Transformers are commonly referred to as transformer encoder-decoders due to their architectural design, which includes both an encoder and a decoder. The encoder is typically used for tasks like text classification, while the decoder is employed for text generation. The encoder processes input data, such as a sentence or amino acid sequence in proteomics, and generates a fixed-size vector containing information about the input. On the other hand, the decoder takes the fixed-size vector and uses it to generate a sequence of words or tokens.

### 3.1 Pre-trained transformer models

Additionally, there are pre-trained Transformer models like TAPE [27], ProtTrans family models (ProtBert-BFD, ProtT5-XL, and ProtT5-XXL) [28], ESM-1, with models with different sizes [29], and ESM2 [30]. All of them were trained for several proteomic tasks, like protein structure prediction, protein function prediction and more. Moreover, recent methods are fine-tuning these pre-trained models for some steps during the process of neoantigen detection like peptide-MHC binding prediction and pMHC-TCR interaction with good performance. In Table 2, we present a detailed comparison of these models.

**Table 2:** Pre-trainned BERT models for several protein tasks: TAPE, ProtBert, ESM1, and ESM-2.

Model	Dataset	Samples	Layers	Hidden size	Attention heads	Parameters
TAPE	Pfam	30M	12	768	12	92M
ProtBert-BFD	BFD	2122M	30	1,024	16	420M
ProtT5-XL	Uniref50, BFD	2122M	24	1,024	32	3B
ProtT5-XXL	Uniref50, BFD	2122M	24	1,024	128	11B
ESM-1 (6 layers)	Uniref50	60M	6	768	12	43M
ESM-1 (12 layers)	Uniref50	60M	12	768	12	85M
ESM-1 (34 layers)	Uniref50	60M	34	1,280	20	670M
ESM-1b	Uniref50	60M	34	1,280	20	650M
ESM-2 (6 layers)	Uniref50	60M	6	320	20	8M
ESM-2 (12 layers)	Uniref50	60M	12	480	20	35M
ESM-2 (30 layers)	Uniref50	60M	30	640	20	150M
ESM-2 (33 layers)	Uniref50	60M	33	1,280	20	650M
ESM-2 (36 layers)	Uniref50	60M	36	2,560	20	3B
ESM-2 (48 layers)	Uniref50	60M	48	5,120	20	15B

Tasks Assessing Protein Embeddings (TAPE) [27] represents the initial effort to assess semi-supervised learning applied to protein sequences. TAPE comprises twelve layers, each containing 512 units and featuring eight attention heads, resulting in a total of 92 million parameters. The authors employed semi-supervised training using the Pfam dataset [31], which encompasses around thirty million protein domains. It is important to note that the Pfam dataset is a subset of the UniProt Knowledge Base (UniProtKB) [32]. Specifically, Pfam utilizes sequences exclusively from the Reference Proteomes [33] within UniProtKB, rather than incorporating the entire UniProtKB collection. Consequently, Pfam contains almost half the number of protein sequences compared to other datasets that are based on the entirety of UniProtKB.

ProtBert-BFD is a member of the ProtTrans family of models, as introduced by Elnaggar et al. [28]. In their study, the authors conducted evaluations using various deep learning architectures on three distinct datasets: BFD, UniRef50, and UniRef100, which encompass 2,122 million, 45 million, and 216 million sequences, respectively. BFD stands out as the most extensive collection of protein sequences, formed by merging data from UniProt [34] and proteins obtained from various metagenomics sequencing projects. On the other hand, UniRef [35] provides a curated set of protein sequences derived from UniProtKB. It is worth noting that the larger dataset, BFD, is known to contain more noise, including sequence errors [28]. Several models were proposed in this context, including ProtBert-BFD, ProtT5-XL, and ProtT5-XXL, boasting 420 million, 3 billion, and 11 billion parameters, respectively. ProtBert-BFD was trained exclusively on the BFD dataset. In contrast, the ProtT5 models underwent training using BFD initially, followed by further training with UniRef50, resulting in performance improvements of 2.8 % for ProtT5-XL and 1.4 % for ProtT5-XXL, respectively. Interestingly, despite the larger parameter size, ProtT5-XL outperformed both ProtBert-BFD and the larger model, ProtT5-XXL. The authors noted that while an increased number of samples did contribute to improved performance, it did not exhibit a consistent similarity to the model size. They proposed that larger models require access to larger datasets, as they tend to see fewer samples when processed within the same computing capacity.

ESM-2 [30] is part of the evolutionary scale of transformer models, ranging from 8 million to a staggering 15 billion parameters. This model is rooted in BERT [24] and manages to outperform its predecessor, ESM-1b [29], by eliminating dropout in both hidden and attention layers. Notably, the authors found that conventional absolute positional encoding methods do not generalize well. Consequently, they turned to Rotary Position Embedding (RoPE) for improved results. Although the use of RoPE slightly increases the training cost, it enhances the model’s quality, particularly for smaller models [30]. Furthermore, for their experiments, the authors utilized the non-redundant UniRef50 dataset from UniProt, which contains an impressive 60 million protein sequence.

## 4 Neoantigen candidates detection

**Figure 3:**
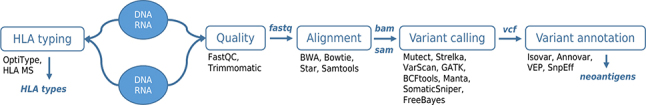
Process for the detection of neoantigen candidates initially, DNA/RNA sequencing is conducted on both tumor and normal cells. Subsequently, quality assessment tools are employed, followed by the utilization of alignment tools. The process then proceeds to variant calling to identify variants. Finally, variant annotation tools are applied to generate a list of potential neoantigen candidates. Moreover, MHC typing tools are employed to determine the HLA or MHC types.

The detection of neoantigens relies on an initial identification of candidates, which is followed by their subsequent prioritization. In this section, we will explain the process of detecting neoantigens candidates (stage 2 in Figure 1b). Detection of neoantigen candidates involves a multi-step process (see Figure 3). Initially, DNA/RNA sequencing is conducted on both tumor and normal cells. Subsequently, quality assessment tools are employed, followed by the utilization of alignment tools. The process then proceeds to variant calling to identify genetic variants. Finally, variant annotation tools are applied to generate a list of potential neoantigen candidates. In addition to the aforementioned steps, quantitative proteomic tools are utilized for mass spectrometry (MS) data analysis. Moreover, MHC typing tools are employed to determine the Human Leukocyte Antigen (HLA) or Major Histocompatibility Complex (MHC) types.

### 4.1 Alignment

The initial stage involves the examination of DNA and RNA sequences obtained from tumor and normal cells. To ensure data quality, standard quality assurance tools such as FastQC or Trimmomatic are typically employed for both RNA-seq and DNA-seq samples. Following quality assessment, alignment tools are applied to align the sequences accurately. In this field, samples from tumor and normal cells are mapping to a reference genome. For this task, there are well-established tools, and Transformers methods are not used according to our systematic search. The most know tools are BWA [36], Bowtie2 [37], and Samtools [38]. Additionally, STAR is one of the most used because it aligns tumor samples more effectively [17]. The output of this stage consists of BAM, SAM alignment files.

### 4.2 Variant calling

Variant calling is the process by which we identify variants from sequence data. This stage, take as input the alignment files of the previous stage (see Figure 3). For variant calling, MuTect [39], Strelka [40], SommaticSniper [41], FreeBayes [42], VarScan [43], and BCFtools [38] are normally employed. Additionally, the information from both methods could be combined, following some approaches [17, 44], [45], [46]. Importantly, there is GATK [47], which integrated other tools and deliver best practices [48, 49] for identifying single nucleotide polymorphism (SNP), and indels in germline DNA and RNA data. Nevertheless, these tools doesn’t used Transformer methods.

Furthermore, there are ongoing efforts to integrate deep learning and Transformer methods. One of the first neural networks is DeepVariant [50], which utilizes a pictorial representation of the local alignment between reads mapping to the site and the segment of the reference sequence surrounding the site (referred to as a pileup); then, convolutional layers are applied. Additionally, there is Clairvoyante [51], which is a convolutional neural network designed to predict variant types. In this case, the authors encode alignment data into images of dimensions 33 × 4 × 4, where 33 corresponds to the position, 4 corresponds to the count of A, C, G, or T; and the third dimension of 4 corresponds to the method of counting. Furthermore, there is Hello [52], which also develops a method leveraging alignment data as images to utilize the Inception-v3 architecture. Moreover, another model has been applied to enhance somatic variant calling through the identification of somatic variants [53]. Another approach utilizes a machine learning classifier to discern between germline and somatic mutations [54]. These initiatives, in collaboration with variant calling tools, have the potential to enhance neoantigen detection; however, further research is imperative for a comprehensive understanding of their effectiveness.

Moreover, the majority of variant calling tools are primarily focused on single nucleotide polymorphisms (SNPs). However, a smaller number of tools, such as Manta [55], MetaSV [56], and Parliament2 [57], have been specifically developed for the detection of structural variants. Nevertheless, a recent thesis was published in which the authors proposed the utilization of Vision Transformers for structural variant identification and genotyping [58]. This innovative approach draws inspiration from the use of images as proposed by DeepVariant [50], thereby opening new possibilities for the application of Transformers in variant calling methodologies.

### 4.3 Variant annotation

In the subsequent step, variant annotation takes place, utilizing VCF-formatted files to derive peptides resulting from these variants. Various tools, such as Isovar [59], Annovar [60], Ensembl’s Variant Effect Predictor (VEP) tool [61], or SnpEff [62], are commonly employed for this task. Typically, these tools search for variants within databases to provide comprehensive annotation of the identified variants. Moreover, a comprehensive benchmarking study [63] was conducted comparing the performance of Ensembl’s Variant Effect Predictor (VEP) tool, Annovar, and Alamut Batch. The investigation utilized a meticulously curated ground-truth set comprising 298 variants. Notably, VEP exhibited the highest precision in variant annotations, attributed to its utilization of the latest gene transcript versions within its algorithm [63].

In order to complement the variant annotation task, fusion genes are promising candidates [64]. Fusion genes, formed by the merging of two or more independent genes, have implications in various cancer types, as evidenced by studies [18, 65], [66], [67], [68], [69], [70]. To enhance neoantigen detection outcomes, integrating annotation tools with fusion gene detection tools is a promising approach. Moreover, FusionGDB [71] serves as an annotation database for human fusion genes. Several tools, such as FusionCatcher [72], Arriba [73], and FusionQ [74], are capable of detecting both novel and known fusion genes. Additionally, there are workflows which include fusion genes detection methods: Integrate-neo [75], neoFusion [76], pVACfuse [77], NeoepitoPred [78], Epidisco [17] and Antigen.garnish [79]. Notably, the use of Transformers in fusion gene detection has not been explored as of now.

### 4.4 HLA typing

Human leukocyte antigen (HLA) serves as the major histocompatibility complex (MHC) for humans, and HLA typing involves identifying specific HLA types such as A03:01 or B07:02, etc. OptiType [80] is a tool designed for HLA typing using RNA-seq data, providing accurate and efficient results in this context. In addition, HLA MS [81] is another tool specifically tailored for HLA typing, utilizing mass spectrometry (MS) data to determine HLA types. This method offers an alternative approach for HLA typing and contributes to the diversity of available techniques in this field. Furthermore, until now, the application of Transformers in the domain of HLA typing has not been explored.

## 5 Neoantigen prioritization

Neoantigen prioritization is the third stage in cancer vaccines development (Figure 1b). This stage takes candidates neoantigens and then predict their affinity to the Major Histocompatibility Complex (MHC), this problem is know as pMHC binding prediction problem. Then, this pMHC complex is used to predict the interaction with the T-cell Receptor (TCR). Both problems takes two protein sequences as input, and the goal is to predict their affinity (regression) or binding (classification). In summary, the proteins can be represented as *p* = {*A*, …, *Q*} and *q* = {*A*, *N*, *K*, *L*, …, *Q*}. Then, we need to know the probability of affinity between *p* and *q*.

### 5.1 Databases

To prioritize neoantigens, researchers often collect samples from various sources, typically drawing from previous studies and similar resources. However, there are publicly available datasets, as listed in Table 3, that specifically focus on the interaction between peptides and MHC (*peptide-MHC*) [82–85], as well as the interaction between pMHC and TCR [86, 87]. Notably, a recent study provides 3D structures of peptides and HLA, introducing a novel avenue of investigation from a different perspective. Finally, the Immune Epitope Database (IEDB) [88] stands out as an exemplary resource in this domain.

**Table 3:** Public databases of pMHC binding, and pMHC-TCR interaction.

Name	Year	Description	Samples	Format	Sample type
VDJdb	2018 [86, 87]	It houses TCR sequences with known antigen specificity and MHC context. Additionally, each record has a confidence score indicating its reliability	5,491	IMGT, IEDB-specific, Kabat, Chothia, Fasta, JSON	TCR sequences
IEDB	2018 [88]	It stores data on epitopes, their associated immunoglobulins (antibodies) and T cell receptors, and how they interact with various immune system components	5 million	IEDB-specific	Both pMHC classes
TSNAdb	2018 [82]	Focuses on tumor-specific antigen mutations, including information on the mutation itself, affected tumor types, and experimental evidence	7,748	TSNAdb-specific	Neoantigens
NeoPeptide	2019 [83]	Specializes in neoepitopes, which are tumor-specific antigens created by mutations. Provides information on predicted immunogenicity and supporting evidence	1 million	NeoPeptide-specific	Stores neoepitopes that could be presented by either pMHC-I or pMHC-II
pHLA3D	2019 [89]	Stores 3D structures of MHC-peptide complexes (key molecules in immune response) along with additional structural and interaction information	106	PDB, mmCIF, pHLA3D-specific	Primarily stores 3D structures of pMHC-I complexes
dbPepNeo	2020 [84]	Similar to NeoPeptide, focusing on neoepitope prediction and immunogenicity, but also offering gene expression data across different tumor types	400,000	dbPepNeo-specific	Both pMHC classes
dbPepNeo2.0	2022 [85]	An updated version of dbPepNeo with more comprehensive neoepitope data, including prediction information, expression data, and additional features	800,000	dbPepNeo/dbPepNeo2.0-specific	Both pMHC classes
IntroSpect	2022 [90]	It is a tool for building databases on pMHC binding. It uses data from Mass Spectrometry	100,000	mzML, mzXML, IntroSpect-specific	Does not directly store pMHC-I or pMHC-II data, but can be used to analyze peptide sequences that might bind to either type
IPD-IMGT/HLA	2022 [91]	Houses information on HLA genes (important for immune response) including allele sequences, nomenclature, and haplotype data	300,000	MGT, FASTA, IMGT-specific, PD-IMGT/HLA-specific	Contains information on HLA genes

### 5.2 pMHC binding prediction

**Figure 4:**
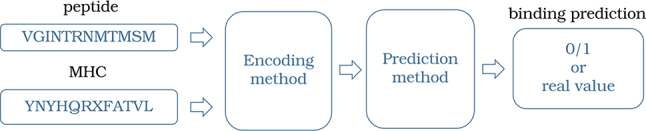
Picturing pMHC binding prediction: initially, the peptide and MHC undergo transformation or encoding into a vector of real numbers. Subsequently, a prediction model is applied, yielding a binary output of 0 or 1 to signify binding or affinity based on real-number predictions.

The prediction of pMHC binding represents one of the final stages in the prioritization of neoantigens. Two fundamental approaches are employed to investigate pMHC bindings: (i) pMHC binding affinity (BA), which assesses the binding preferences of peptides and MHC [92]; and (ii) MHC eluted ligands (EL), generated through Liquid Chromatography Mass Spectrometry (LC-MS), enabling the identification of a large number of eluted ligands in a single experiment [93]. However, both methodologies are characterized by time-intensive and expensive processes, prompting the emergence of computational methods for predicting pMHC bindings. The methodology used is depicted in Figure 4, where two amino acid chains *p* = {*a*, *w*, *d*, *r*, *a*, *b*, …} and *q* = {*b*, *w*, *c*, *x*, *a*, *r*, …} are taken as inputs; then the amino acid are encoded or transform into a vector of real numbers (in Table 4, we described encoding methods); then, a prediction model is applied, yielding a binary output of 0 or 1 to signify binding or affinity based on real-number predictions.

**Table 4:** Description of amino acid encoding types used in pMHC binding prediction and neoantigen prioritization.

Amino acid encoding	Reference	Description
BLOSUM62	[94–97]	Type of BLOSUM matrix
BLOSUM50	[98, 99]	Type of BLOSUM matrix
One-hot	[27, 28, 100], [101], [102], [103], [104]	Binary vector assigns unique index to each amino acid, marking specific amino acid with a one
One-hot and BLOSUM	[105–108]	A combination of one-hot and BLOSUM
Universal google encoder	[109]	Encodes text for tasks like classification, similarity, and clustering using high-dimensional vectors
AAindex	[110, 111]	A database of numerical indices representing physicochemical and biochemical properties of amino acids
3D amino acid	[112]	3D coordinates of each amino acid
Physicochemical properties	[113–115]	Physicochemical properties of individual amino acids like: pH, charge, isoelectric point, and stereometric structure

The approaches for predicting pMHC binding can be broadly classified into two categories: allele-specific and pan-specific methods. Allele-specific methods involve training a distinct model for each specific allele, while pan-specific methods entail the training of a universal model applicable across a range of alleles. Moreover, there two types of MHC related to immunology: MHC class I and MHC class II. Both classes of proteins serve the common function of presenting peptides on the cell surface for recognition by T cells. pMHC-I complexes are exhibited on nucleated cells and are identified by cytotoxic CD8+ T cells. Conversely, the presentation of pMHC-II by antigen-presenting cells, such as dendritic cells (DCs), macrophages, or B cells, can activate CD4+ T cells [116].

Furthermore, one of the most widely utilized metrics for evaluating pMHC binding predictions is the Area Under the ROC Curve (AUC). The Receiver Operating Characteristic (ROC) curve is a graphical representation illustrating the performance of a classification model at various classification thresholds. AUC serves as a comprehensive metric, offering an aggregated measure of performance across all conceivable classification thresholds. This metric is widely employed due to the dynamic nature of threshold selection in machine learning methods for predicting binding, which varies based on distinct peptide lengths and MHC types. Additionally, the Spearman’s rank correlation coefficient (SRCC) is utilized. This metric offers a comprehensive analysis of crucial factors influencing performance comparison across various positive and negative sample ratios [117].

An innovative pan-specific approach for pMHC binding prediction is presented by NetMHCpan [118]. This method employs a traditional feed-forward network with a single hidden layer and one output neuron. The authors conducted extensive testing by varying the hidden layer size, exploring configurations ranging from 22 to 86 neurons. Additionally, the authors introduced the concept of pseudo sequences for MHC proteins, which are composed of amino acid residues in direct contact with the peptide. This novel approach enhances the predictive capabilities of the model, providing a more comprehensive understanding of pMHC-I interactions.

Since then, subsequent iterations such as NetMHCpan2.0 [119], NetMHCpan3.0 [120], and NetMHCpan4.0 [121] have been developed. The version, NetMHCpan4.0, incorporates information from binding affinity (BA) and mass spectrometry (MS) ligands. Notably, the authors introduced the NNAlign training approach, enabling combined training on BA and MS EL data. In their methodology, an ensemble of 40 feed-forward networks was employed, with each network featuring a hidden layer comprising 60 to 70 neurons. Furthermore, the encoding of each amino acid was achieved using the BLOSUM matrix. This sophisticated approach enhances the predictive accuracy of the model by integrating diverse sources of information and employing an ensemble strategy for training.

In 2020, the latest iteration, NetMHCpan4.1, was introduced [16], maintaining the same network architecture as its predecessor. However, a significant enhancement was made by expanding the dataset to include eluted ligands multiple allelic (EL-MA) data. EL-MA data are originated from mass spectrometry (MS) experiments, where EL assays exhibit polyspecificity, associating one peptide with multiple alleles. The incorporation of EL-MA data introduces complexities in analysis and interpretation [122], requiring the use of algorithms know as deconvolution to transform these EL-MA data into individual pMHC pairs. NetMHCpan4.1 implemented NNAlign_MA, a modification of NNAlign, specifically designed to accommodate EL-MA data. This adaptation reflects the methodological evolution necessary to handle the unique challenges posed by EL-MA datasets. Presently, NetMHCpan4.1 stands as the benchmark method in the field, showcasing its adaptability and effectiveness in addressing the intricacies of pMHC-I prediction [16].

To summarize, the challenge of pMHC binding prediction has prompted the application of various machine learning methods like support vector machines (SVM), shallow neural networks (SNN), and random forest (RF). Moreover, this review specifically concentrates on approaches based on Transformers. In the subsequent section, we will delve into the details of these Transformer-based methods. In Table 5 we provide a comprehensive comparison of these Transformer models and deep learning methods that incorporate attention mechanisms.

**Table 5:** Transformers and deep learning methods with attention mechanism used for pMHC binding prediction.

Year	Name	Input	Model
2023 [123]	ESM-GAT	One-hot	BERT with transfer learning from ESM1b and ESM2 fine-tuned with a graph attention network (GAT) at the end. It outperformed NetMHCpan4.1
2023 [124]	CapsNet-MHC	BLOSUM62	Capsule neural network, it outperformed state-of-art tools for small peptides of 8 to 11-mer
2023 [125]	STMHCpan	One-hot	A star-transformer model, it use usefull for anylenght peptides and could extended for predicting T-cell responses
2023 [126]	DapNet-HLA	Fused word embedding	Combined the advantages of CNN, SENet (for pooling), and LSTM with attention
2022 [127]	HLAB	One-hot	BERT from ProtBert pre-trained model followed by a BiLSTM with attention mechanism
2022 [103]	MHC RoBERTa	One-hot	RoBERTa pre-trained and followed by 12 multi-head SA and a FC layers, it outperformed NetMHCPan 3.0
2022 [104]	TransPHLA	One-hot	It used SA mechanism based on four blocks, it slightly outperformed NetMHCpan4.1 and is faster making predictions
2021 [102]	CapTransformer	One-hot	Transformer with cross attention pooling to capture local and global information
2021 [101]	ImmunoBERT	One-hot	BERT from TAPE pre-trained followed by a linear layer. Authors claimed that N-terminal and C-terminals are highly relevant after analysis with SHAP and LIME
2021 [100]	BERTMHC	One-hot	BERT from TAPE pre-trained followed by a linear layer. It outperformed NetMHCIIpan3.2 and PUFFIN
2021 [95]	MATHLA	BLOSUM	It integrates BiLSTM with multi-head attention. It achieved an AUC score of 0.964, compared to 0.945, 0.925 and 0.905 for netMHCpan 4.0, MHCflurry and ACME respectively
2021 [105]	DeepSeqPanII	BLOSUM62 and one-hot	It has two LSTM layers, an attention block and three FC layers. It got better results than NetMHCIIpan 3.2 on 26 of 54 alleles
2021 [98]	DeepNetBim	BLOSUM50	It uses separate CNNs for pMHC binding and immunogenetic with a attention module. It got 0.015 MAE for binding and 94.7 of accuracy for immunogenic
2021 [94]	DeepAttentionPan	BLOSUM62	CNN with an attention mechanism. It is allele-specific and got slightly better results than ACME for allele level
2021 [128]	SpConvM	One-hot, BLOSUM, and deep	1D layer of CNN, an attention layer and a FC layer. Moreover, they employed global kernels to enhance their results, along with a combination of onehot, BLOSUM, and deep
2020 [129]	MHCAttNet	One-hot	CNN followed by an attention layer to generate a heat map over the amino acids
2019 [99]	ACME	BLOSUM50	CNN with attention, it extract interpretable patterns about pMHC binding. Moreover, it got SRCC of 0.569, AUC of 0.9 for HLA-A and 0.88 for HLA-B
2019 [130]	DeepHLApan	One-hot	Allele-specific model with three layers of bidirectional GRU (BiGRU) with an attention layer. It got acc >0.9 on 43 HLA alleles

#### 5.2.1 CNN with attention

There are Convolutional Neural Network (CNN) models that incorporate an attention mechanism, such as ACME [99]. ACME utilizes a CNN with an attention module that assigns weights to individual residue positions, aiming to assign higher weights to residues of greater importance in pMHC interactions. ACME achieved a Spearman Rank Correlation Coefficient (SRCC) of 0.569, which is higher than NetMHCpan 4.0. Next is MHCAttNet [129], which uses a CNN followed by an attention layer. The attention layer is used to generate a heat map over the amino acids, indicating the important subsequences present in the amino acid sequence. Another CNN-based model is DeepAttentionPan [94], which uses deep CNN to encode peptides and MHC into vectors of dimensions 40 × 10 × 11 before employing an attention module to calculate positional weights. We also have DeepNetBim [98], which incorporates an attention module similar to ACME and DeepAttentionPan. However, it uses two separate CNNs to predict pMHC binding and immunogenicity, which are later combined in the final layers. Furthermore, in their study on SpConvM [128], the authors demonstrated that incorporating global kernels into CNN with attention yielded superior performance. Additionally, their experiments involved a comparison of different amino acid encoding methods, including onehot, BLOSUM, and Deep. According to their findings, the combination of onehot, BLOSUM, and Deep together resulted in improved outcomes. Recently, the use of Capsule Neural Networks (CapsNet) has emerged to model hierarchical relationships. CapsNet-MHC [124] is proposed to predict pMHC-I binding, and it outperformed other tools like HLAB, ACME, Anthem, and NetMHCpan4.1 for small peptides of 8–11-mers.

#### 5.2.2 RNN with attention

Additionally, several recurrent neural networks (RNNs) have been introduced, such as DeepHLApan [130], which is an allele-specific model that considers pMHC binding and immunogenicity data. The model features three Bidirectional Gated Recurrent Unit (BiGRU) layers and an attention layer, ultimately outputting the binding and immunogenicity scores. Moreover, this approach incorporated CD8+ T-cell epitopes and Mass Spectrometry data and achieved an accuracy exceeding 0.9 for 43 HLA alleles. Furthermore, the allele-specific model DeepSeqPanII [105] utilized a combination of BLOSUM62 and one-hot encoding, with a specific focus on MHC-II. The model included two layers of Long Short-Term Memory (LSTM) networks with 100 hidden units and an attention block to extract weighted information based on hidden units. The attention block consisted of four 1-D convolutional layers, and three fully connected layers were employed to predict affinity. DeepSeqPanII outperformed NetMHCIIpan 3.2 for 26 out of 54 alleles. Another RNN is MATHLA [95], which used a Bidirectional Long Short-Term Memory (BiLSTM) to learn dependencies among amino acid residues and applied multiple-head attention to acquire positional information for the output of BiLSTM. The output was further processed through 2-D convolutional layers. MATHLA achieved an AUC score of 0.964, surpassing the performance of NetMHCpan 4.0, MHCflurry, and ACME, which scored 0.945, 0.925, and 0.905, respectively. Recently, the allele-specific DapNet-HLA [126] introduced an additional dataset from Swiss-Prot for negative samples. The method used an embedding block for each token and its absolute position, which was compared against several encoding techniques, including Dipeptide Deviation from Expected mean (DDE), Amino Acid Composition (AAC), Dipeptide Composition (DPC), and Encoding based on Grouped Weight (EGBW). Recently, DapNet-HLA combined the advantages of CNN, SENet (for pooling), and LSTM, achieving high scores, although it was not directly compared to state-of-the-art methods.

#### 5.2.3 Transformers

BERTMHC [100] was one of the pioneering works to incorporate the BERT architecture. This pan-specific pMHC-II binding/presentation predictor employed transfer learning from Tasks Assessing Protein Embeddings (TAPE) [27], a model trained with data from the Pfam database comprising thirty-one million proteins. The authors integrated average pooling followed by a fully connected (FC) layer after the TAPE model. According to BERTMHC’s experiments, BERTMHC outperformed NetMHCIIpan3.2 and PUFFIN, achieving an AUC of 0.8822 compared to 0.8774. Similarly, ImmunoBERT [101] leveraged transfer learning from TAPE, focusing on pMHC-I prediction. The model involved stacking a classification token’s vector after the TAPE model. The authors’ analysis concluded that amino acids in proximity to the peptide N/C-terminals are of high relevance, with positions in the A, B, and F pockets assigned high importance, as determined by LIME and SHAP analyses. Additionally, CapTransformer [102], introduced an innovative cross-attention pooling mechanism that effectively aligns and aggregates peptide-MHC residue features jointly. By utilizing both self-attention and cross-attention, it facilitates the learning of feature representations for individual residues and global binding information, resulting in superior performance compared to NetMHCpan4.0.

Other methods that utilized transfer learning include MHCRoBERTa [103] and HLAB [127]. The first one, employed five encoders with twelve multiple-head self-attention mechanisms. Initially, the approach utilized self-supervised training with data from the UniProtKB and Swiss-Prot databases. The method also applied sub-word tokenization and outperformed NetMHCpan4.0 and MHCflurry2.0, achieving an SRCC of 0.543. HLAB leveraged transfer learning from ProtBert-BFD [28], which was trained with data from the BFD dataset containing 2,122 million proteins. HLAB employed a BiLSTM model and achieved superior performance to state-of-the-art methods, including NetMHCpan4.1. Moreover, an additional research examined the application of transfer learning and padding [131]. Finally, TransPHLA [104] is an allele-specific method that applies self-attention to peptides. The model consists of four modules: an embedding block, an encoder block with multiple self-attention mechanisms, a feature optimization block (utilizing FC layers), and a projection block (employing FC layers for prediction). TransPHLA has outperformed state-of-the-art methods, including NetMHCpan4.1, and offers the advantage of effectiveness for peptides and MHC alleles of varying lengths.

An interesting proposal involve the use of Star-Transformer, SMHCpan [125], a lightweight model where the FC structure is replaced with star shaped topology. Moreover, Graph Neural Networks (GNN) have been used in several Protein-Protein Interaction (PPI) problems because they manage protein relations. In this context, a Nobel proposal ESM-GAT [123] arose, which used BERT architectures and transfer learning from ESM1b and ESM2 models, then it stacked a Graph Attention Network (GAT). It outperformed NetMHCpan4.1; nevertheless, the authors didn’t compare their proposal with other state-of-art tools.

### 5.3 pMHC interaction with TCR

TCRs consist of chains *α* and *β*, both chains contain information about the specificity of the TCR to determine the binding prediction with pMHC. Each chain has three loops called Complementarity Determining Regions (CDRs) are responsible for binding TCR and pMHC, there are three regions; region 1 and 2 are very likely to bind to pMHC, therefore region 3 is the determining factor for the prediction.

Models, in general, have as input TCR and pMHC sequences. Representing these data and feature extraction are essential for achieving better results. Many models use the BLOSUM MATRIX, while some models employ one-hot encoding, and others combine BLOSUM and one-hot encoding. However, some opt to used Granularity vectors [132]. The dimensionality of the input may vary depending on the chain or region, we must take into account the way it is encoded to extract the characteristics. There are several studies that use multi-head self-attention, recurrent layers and convolutional layers (see Table 6).

**Table 6:** Transformers and deep learning methods with an attention mechanism used for pMHC interaction with TCR.

Year	Name	Input	Model
2023 [133]	diffRBM	BLOSUM62	For the model of immunogenicity allele-specific presentation employs models with an RBM architecture transfer learning and restricted Boltzmann machines
2023 [134]	AVIB	BLOSUM50	Make use of attention of experts (AoE). Leverages multi-head self-attention to predict the interactions between TCRs and peptides
2023 [135]	BERTrand	BLOSUM62	Employs a BERT model with 8 transformer blocks and over 2.5 M parameters, to compensate the number of parameters uses an unsupervised pre-training strategy
2023 [136]	Hybrid gMLP	BLOSUM50	gMLP based on deep learning with multiple attention mechanisms to predict the interaction of MHC and TCR peptide, can handle the problems caused by different TCR lengths
2023 [137]	PiTE	BLOSUM	Uses an amino acids embedding model and a sequence encoder. The representations concatenated with their absolute subtraction are fed to two linear layers
2023 [138]	MIX-TPI	BLOSUM62	It employs CNNs whit self-attention to construct a sequence-based and a physicochemical-based extractor. Then it fuses them with a self-attention layer to predict TCR–pMHC interactions
2023 [139]	TCRdock	BLOSUM62	A specialized version of AlphaFold to generate models of TCR:peptide-MHC interactions that can be used to discriminate correct from incorrect peptide epitopes
2022 [140]	ATMTCR	BLOSUM50	ATMTCR feds into two fully-connected layers a contrastive learning-based model and NetMHCpan to predict the binding of TCR and pMHC complex
2022 [141]	ATM-TCR	One-hot & BLOSUM	Consists of two encoders and a linear decoder. Each of sequences are aligned via IMGT. Calculates the similarity of the attention maps and reference maps to confirm if it is a binding
2022 [132]	AttnTAP	Granularity vectors	Dual-input deep learning network that included bi-directional LSTM, attention mechanism and multilayer perceptron to extract TCR and peptide features predict TCR-peptide binding
2022 [142]	pMTattn	Embedding	Employs cross-attention mechanism to learn interaction information between pMHCs and TCRs. To encode each of them into an embedding, it uses transformer-based models
2021 [143]	DLpTCR	One-hot	DLpTCR consits of FCN, LeNet-5 and ResNet-20 for predicting the peptide-CDR3*α*(*β*) and a multi-model ensemble strategy. It also implements an attention mechanism in ResNet-20
2020 [144]	TcellMatch	BLOSUM50	Employs embeddings for amino acids and a sequence embedding block composed by multiple layer types. The activation generated is fed into a dense network to predict binding events

#### 5.3.1 CNN with attention

Convolutional neural networks (CNNs) with attention are employed because they effectively capture and process the data. MIX-TPI [138], a multimodal computational framework, utilizes CNNs with self-attention. CNNs are utilized to construct sequence-based extractors (SE) and physicochemical-based extractors (PE), which are responsible for learning refined sequence and physicochemical features, respectively. Self-attention is employed to fuse these representations for predicting TCR–pMHC binding.

#### 5.3.2 RNN with attention

Recurrent neural networks (RNN) perform tasks involving sequential amino acid data from TCR and pMHC, there are several studies RNNs with attention mechanisms, such as DLpTCR [143] and AttnTAP [132]. DLpTCR is a set of three-architecture deep learning include FCN, LeNet-5 and ResNet-20 for predicting the likelihood of peptide-TCR interaction, used attention mechanism in ResNet-20 to improve the quality of the generated outputs. AttnTAP is a dual-input deep learning framework including bi-directional long short term memory (BiLSTM) and multi-layer perceptron (MLP), and Attention mechanism to extract TCR and peptide features separately and perform TCR-peptide binding prediction.

Some studies utilized CNN, RNN and attention as TcellMatch [144]. TcellMatch is a set of multi-architecture deep learning models, used a newly technology named single-cell, this technology enables the simultaneous sequencing of TCR chains *α* and *β* and determining the T-cell specificity. This study, also makes multiple comparisons and demonstrates that models that include both the *α* chain and the *β* chain have a predictive advantage over models that only include the *β* chain, although, the difference is short, but significant.

#### 5.3.3 Transformers

At present, a variety of models based on transformers have been developed. ATM-TCR [141] introduced the use attention mechanisms as a main part, present a model which uses multi-head self-attention network. This model consists of two encoders for TCR and epitope sequences and a linear decoder for determining the binding, used a multi-head self-attention network to obtain contextual representations of each sequence, each of the TCR and epitope sequences are aligned via IMGT and used the euclidean distance to calculate the binding or not binding. AVIB [134] a multi-sequence generalization of Variational Information Bottleneck, introduced a novel method Attention of Experts (AoE). AoE can take advantage of the abundant available data where the CDR3*α* chain or CDR3*β* chain sequence is missing when estimating the multi-sequence variational posterior. AVIB utilized AoE to implicitly estimate the posterior distribution of latent encodings, taking into account multiple input sequences. This model can handle missing data sequences at test time, also takes leverages multi-head self-attention to predicting the interactions between TCRs and peptides. BERTrand [135] trains a peptide-TCR binding model with a degree of cross-peptide generalization. The architecture, BERT, has been previously pretrained and fine-tuned for peptide-TCR classification. Initially, peptides and TCRs are represented as sequences of tokens, and each peptide and TCR are concatenated into a unified sequence that preserves their positional and typological information. This composite sequence, along with its positional and typological embeddings, is then fed into a BERT network comprising 8 transformer blocks. The resulting output is subsequently directed to both a token classification head and a sequence classification head during the pre-training and fine-tuning phases. Hybrid gMLP [136] combined gMLP model with the attention mechanism, where the information was obtained by gMLP, and then Multi-head Attention and Local-Attention were used to extract the correlation information of the CRD3 TCR-pMHC. This framework can handle the problems caused by different TCR lengths. Additionally, demonstrated that the models trained with paired CDR3-chain *α* and CDR3-chain-chain *β* data are better than those trained with only CDR3-chain-chain *α* or with CDR3-chain-chain *β* data. This model may be potential, but it lacks a large database to input its powerful model. TCRdock [139] is a especialized version of neural network predictor AlphaFold, show that predictor AlphaFold can be used to discriminate correct from incorrect peptide epitopes with substantial accuracy. Additionally valuated structural modeling as a potential avenue for prediction of TCR epitope specificity.

PiTE [137] a two-step pipeline for the TCR-epitope binding prediction. Firstly, amino acids embedding model pre-trained and second binding affinity prediction model that consists of two sequence encoders and a stack of linear layers. This study makes a benchmarking several types of neural network architectures for sequence encoders usable in existing models, including Average Pooling (Baseline), BiLSTM, CNNs and Transformers. Average Pooling helps to reduce the dimensionality of the inclusion of amino acids whose size is generally larger than the inclusion of BLOSUM, in Transformers select a multi-head self-attention module for sequence encoders allowing the model to attend to different amino acid residues of a sequence, BiLSTM structure as sequence encoders because it can learn features from both directions and designs an CNN-based architecture for the sequence encoders using ByteNet. Demonstrating that the Transformer-based method achieved the best performance.

Other methods that utilized transfer learning include diffRBM [133] and pMTattn [142]. DiffRBM is a sequence-based approach using transfer learning and Restricted Boltzmann Machines (RBM), RBM is mainly used in data representation and generation. This approach relies two different sets of data generically as ‘selected’ and ‘background’ datasets of large size, both sets are trained whit RBM model. pMTattn was one of the pioneering model in adopt a cross attention mechanism, allowing you to focus on important binding sites. pMTattn takes advantage of transfer learning and an attention mechanism, was one of the pioneering model in adopt a cross attention mechanism, allowing you to focus on important binding sites, therefore, the information on the binding site of TCR and pMHC is completely known.

## 6 Bioinformatic pipelines and clinical trials

As elucidated in preceding sections, the process of neoantigen detection entails a multifaceted workflow, commencing with the identification of neoantigen candidates and culminating in their prioritization, as illustrated in Figure 1b. To navigate this intricacy, researchers have devised pipeline tools, encapsulating various tools essential for neoantigen detection. Subsequent to the *in silico* detection of neoantigens facilitated by these pipeline tools, the subsequent phases involve *in vitro* vaccine development and clinical trials. Hence, this section delves into the utilization of pipeline tools for neoantigen detection and highlights clinical trials which assessed the efficacy of neoantigen vaccines.

### 6.1 Bioinformatic pipelines

A bioinformatic pipeline in the neoantigen context is a software construct that assembles various command line tools. In the realm of neoantigen detection and prioritization, reliance on multiple tools is essential. For instance we can use tools such as (1) FastQC ensure sequence quality, (2) BWA handles alignment, (3) Samtools manipulates BAM files, (4) BCFtools is employed for variant calling, (5) Annovar provides variant annotation, and (6) netMHCpan4.1 predicts pMHC binding and pMHC-TCR binding affinity. However, the use of these diverse tools can introduce compatibility and dependency challenges. To address this issue, developers have created pipeline tools aimed at enhancing the usability of neoantigen detection software. These pipelines effectively manage the integration of these tools, mitigating potential conflicts and dependencies, thereby streamlining the overall neoantigen analysis process.

In Table 7, we present pipelines published since 2018. These pipelines use various types of information as input. For instance, the PGV Pipeline [17] and PEPPRMINT [44] use DNA-seq, while other tools such as PGNneo [145], NAP-CNB [146], NaoANT-HILL [147], ProGeo-neo [148], ScanNeo [149], and Neopepse [14] use RNA-seq because these sequences better capture information about mutations and non-coding regions of DNA [145].

To reduce the complexity of pipelines, some proposals have opted to use Variant Calling Format (VCF) as input. These files contain mutation information and are obtained through alignment and mutation calling methods (stages 2.1 and 2.2 in Figure 1b). Tools like HLA3D [150], Neoepiscope [18], pVACtools [77], and NeoPredPipe [151] thus reduce the number of tools used in neoantigen detection. However, the results obtained may be inferior compared to tools that use DNA-seq and RNA-seq.

Additionally, for accurate neoantigen detection, it is necessary to have the sequencing of Major Histocompatibility Complex (MHC) or Human Leukocyte Antigens (HLA) proteins. These proteins are necessary because they are used to predict the binding between potential neoantigens and MHC (pMHC: stage 3.1 in Figure 1b). These proteins are encoded by highly polymorphic genes, leading to substantial variation in peptide (neoantigen) binding, thereby influencing the set of peptides presented to T-cells [152]. In this context, the NeoPredPipe [151], and Neopepsee [14] pipelines request these HLA proteins as input, while others predict this information from DNA-seq. From a usability standpoint, obtaining the HLA types entails unnecessary effort for the user.

As we mention before, fusion genes are related to several types of cancer [18, 65], [66], [67], [68], [69], [70]. Thus, there are pipelines which include fusion genes detection methods: Integrate-neo [75], neoFusion [76], pVACfuse [77], NeoepitoPred [78], Epidisco [17], TrueNeo [153] and Antigen.garnish [79]. Gene fusions typically yield a higher number of neoantigens per mutation compared to single nucleotide variants (SNVs) and insertions/deletions (Indels). Furthermore, fusion-derived neoantigens exhibit heightened immunogenicity. Notably, neoantigens arising from frameshift fusions or passenger fusions are anticipated to possess the greatest immunogenic potential [64].

Furthermore, the type of variant is closely associated with specific cancer types [64]. For instance: (1) SNV are related to Melanoma, Lung cancer and Glioblastoma. (2) Indels are related to microsatellite instability-high tumors, Clear cell, papillary, and chromophobe renal cell carcinomas. (3) Fusion genes are related to Hematologic malignancies, sarcomas, prostate cancer, head cancer and neck cancer. Hence, depending on the cancer type, it is advisable to prioritize the selection of the appropriate analytical pipeline for genetic analysis and interpretation.

**Table 7:** Bioinfomatics pipelines developed for the detection of neoantigens.

Name	Year	Input	Output	Tools
PEPPRMINT	2023 [44]	DNA-seq	Neoantigens	BWA, Mutect, Strelka, ANNOVAR, OptiType, PEPPRMINT, netMHCpan4.1
PGNneo	2023 [145]	VCF, RNA-seq, MS data	Neoantigens	Trimmomatic, BWA, SAMtools, GATK, Picard, OptiType, Annovar, Bedtools, MaxQuant, NetMHCpan4.1, Blastp
HLA3D	2022 [150]	VCF, HLA, SMG, HBV	Neoantigens	MHCcluster, SAVES, PROCHECK, CoDockPP, Verify 3D, ERRAT, ClusterW2, 3Dmol, PSRPRED4.0, MHCf lurry
NextNEOpi	2022 [154]	WES/WGS, RNA-seq	Neoantigens	OptiType, pVACseq, NetMHCpan, MHCflurry, NeoFuse, MiXCR
Seq2Neo	2022 [155]	WES/WGS, RNA-seq	Neoantigens	Mutect2, STARFusion, ANNOVAR, Agfusion, NetMHCpan, MHCflurry, Pick-Pocket, NetMHCcon, TPMcalculator, NetCTLpan
NAP-CNB	2021 [146]	RNA-seq	Neoantigens	Star, Picard, GATK, SplitNCigarsReads, MuTect2, Cufinks, Epi-Seq, pVAC, seq, Neoantimon, MuPeXI, BLOSUM62
NeoANT-HILL	2020 [147]	RNA-seq, VCF	Neoantigens, GE	GATK, Mutect2, Optitype, NetMHC, NetMHCpan, NetMHCCcons, NetMHCstapan, PickPoket, SMM, SMMPMBEC, MHCflurry, NetMHCIIpan, NN-align, SMM-align, Sturniolo, Kallisto
Neoepiscope	2020 [18]	VCF, BAM	Neoantigens	BWA, Bowtie2, Pindel, MuSE, RADIA, SomaticSniper, VarScan2, GATK, HapCUT2
OpenVax	2020 [156]	DNA-seq, RNA-seq	Neoantigens	GATK 3.7, STAR, MuTect 1.1.7, Mutect 2, Strelka, NetMHCpan, NetMHCCcons, SMM, SMM with a Peptide
ProGeo-neo	2020 [148]	RNA-seq, VCF	Neoantigens	SRA Toolkit, BWA, GATK, Bcftools, ANNOVAR, Kallisto, OptiType, NetMHCpan4.0
pVACtools	2020 [77]	VCF	Neoantigens	CWL36, Cromwell37, ADNc38, BWA-MEM25, HaplotypeCaller28, MHCflurry14, MHCnuggets15, NetChop17, INTEGRATE-Neo19
TruNeo	2020 [153]	DNA-seq, RNA-seq	Neoantigens	BWA, GATK v3.3, Somatic SNVs, STAR v2.5.3a, RSEM v1.3.0, NetMHCPan 3.0, netChop
NeoPredPipe	2019 [151]	VCF, HLA	Neoantigens, VA	ANNOVAR, POLYSOLVER, netMHCpan, PeptideMatch
ScanNeo	2019 [149]	RNA-seq	Neoantigens	HISAT2, BEDTools, BWA-MEM, pVAC-Seq, NetMHC, NetMHCpan
Neopepsee	2018 [14]	RNA-seq, VCF, HLA	Neoantigens, GE	NetCTLpan, Swiss-Prot
PGV pipeline	2018 [17]	DNA-seq	Neoantigens	BWA-MEN, BQSR, MuTect, Strelka, STAR, seq2hla, Vaxrank, Isovar, MHCtools, Varcode, pyEnsembl

GN: gene expression, VA: variant annotation, WEG: whole exome sequencing, WGS: whole genome sequencing.

Moreover, neoantigen pipelines deliver its results and demonstrated its performance in several ways. For instance: pVACtools, reported 8-fold increased in the number of strong binding compared to Integrate-Neo. TrueNeo evaluated its performance detecting 19 identified neoantigens from 1,599 non-redundant SNVs from 134 patients. Neverthleses, TrueNeo, compared its performance varying the pMHC tools used (NetMHCpan, MHCflurry, deepHLA, etc.). On the other hand ProGeo-neo, evaluated its performance by applying similarity analysis between 746 validated neoantigens from dbPepNeo2.0 and 6,400 random peptides. These methods of evaluation difficults a comparative analysis of neoantigen pipelines.

### 6.2 Clinical trials

Before applying any type of treatment, there are several stages that any drug, product, or even a specific technique being considered for therapy must undergo. The most basic stage involves conducting preclinical trials. Preclinical trials are studies conducted in laboratories and on animals to assess the safety and efficacy of new treatments before testing them on humans. These trials provide crucial data about potential side effects and determine the appropriate dosage [157, 158]. As for clinical trials, they are investigations carried out in humans to evaluate the safety and efficacy of new treatments or therapies. They are divided into four phases: (1) Phase I: Evaluates safety and determines the initial dosage in a small group of volunteers. (2) Phase II: Focuses on efficacy and continues to assess safety in a larger group of participants. (3) Phase III: Confirms efficacy and monitors side effects in a large population. Compares the new treatment with existing standards. (4) Phase IV: Conducted after approval, continues monitoring safety and long-term effectiveness in real-world conditions. These trials are essential to ensure that new treatments are safe and effective before being applied on a larger scale. In Table 8, a list of recent clinical trials are presented.

Regarding clinical trials of immunotherapy with vaccines based on tumor neoantigens, the subject of this work, comparing results to draw conclusions based on overall survival and disease-free time across different types of neoplasms is not possible due to the heterogeneity of their characteristics, which confer distinct evolution and more or less aggressive behavior. Even comparisons within the same types of neoplasms, referring to the macroscopic aspect (those affecting the same organ), become complicated due to histological and, more importantly, molecular characteristics defined by the types of mutations, leading to different outcomes [159]. This is precisely why current trends in treatment are more focused on the molecular aspect rather than the organ affected by cancer. It is observed today that applying the same drug for cancers affecting very different organs but sharing common mutations can be an effective strategy. This highlights the importance of targeting treatments to molecular targets present in mutations, which is the foundation of immunotherapy based on tumor neoantigens [160]. This type of intervention was applied in the clinical trials analyzed in our work, from which we have derived specific conclusions mentioned below after the analysis.

In all the reviewed clinical trials that have been concluded to date, a common point found is that adoptive cell therapy is safe, resulting in manageable side effects, and generates an immune response effective enough to assist in combating different types of cancer. It also positively contributes to other types of treatments, especially the use of checkpoint inhibitors [161–166]. This is reflected in the increase in disease-free time or the overall survival rate of the patients who participated in these studies compared to conventional treatments. This, of course, depends on the type of neoplasm, the stage it is in, and the more or less aggressive nature of each neoplasm. Particularly relevant is the result of a randomized clinical trial showing that personalized vaccines based on dendritic cells loaded *in vivo* with tumor neoantigens demonstrated generating stronger immune responses with fewer side effects than other types of adoptive cell therapy, specifically tumor cell vaccines exposed to antigens [167].

Furthermore, in all clinical trials, personalized vaccines based on neoantigens were administered to patients with solid tumors, and in most studies, to patients in advanced stages of the disease [100, 161], [162], [163, 165, 167], [168], [169], [170], [171], [172], [173], [174] considering an advanced stage as one in which there is metastasis or extension of the disease. This does not rule out the possibility that this type of treatment could also be applied to hematological cancers.

Lamentably only two of the reviewed studies were randomized [164, 167], all the studies are interventional, demonstrating the researchers’ intent to establish the safety, efficacy, or both parameters for personalized vaccines based on neoantigens, either as individual therapy or in combination with other types of treatments, as previously discussed.

Moreover, close to half of the studies are phase II trials [100, 163, 167, 168, 170], [171], [172, 174], [175], [176], [177], [178] documenting the immunogenic efficacy of personalized vaccines based on tumor neoantigens in the treatment of various types of cancer. It is interesting to note that neoantigens could have other applications not only therapeutically against cancer but also as predictors of the response to immunotherapy treatments such as checkpoint inhibitors, which could be defined by analyzing their interaction with CD8 T lymphocytes [163].

A clinical peculiarity of pancreatic cancer is that it is generally diagnosed in advanced stages, leading to very limited survival post-diagnosis. This is due to its histological properties, allowing it to form a fibrotic barrier that prevents the entry of drugs or their active components into the tumor. Hence, clinical trials for this type of cancer seemingly involved patients who could be diagnosed at earlier stages [166, 179, 180]. A common exclusion criterion in many trials with this type of immunotherapy is the overall poor health of patients, a constant in advanced pancreatic cancer. These pancreatic cancer characteristics limit the progression of clinical studies for various therapeutic approaches, particularly neoantigen-based vaccines. While establishing the safety of the treatment, further studies are needed to determine efficacy or strategies to achieve it. Similar challenges are observed in cancers affecting the central nervous system, where the complexity of the affected organ likely determines a sluggish clinical behavior and evolution in most cases. This impedes conducting clinical trials in advanced stages, and the inherent limitations result in scarce trials, making it challenging to conclusively establish efficacy [181, 182].

In gastrointestinal and hepatic cancers, neoantigen vaccines have been tested in both advanced disease stages and earlier stages [168], [169], [170, 175]. This variation may be attributed to the disease progression depending on the affected organ, as well as the histological and molecular characteristics of each tumor. A preventive vaccine trial was conducted in patients with Lynch syndrome, a hereditary syndrome increasing the likelihood of stomach or colorectal cancer. The trials not only established safety but also demonstrated the efficacy of the treatment [176].

For lung cancers, clinical trials were conducted in advanced disease stages. Safety and efficacy were established, likely linked to the availability of new targeted therapies, contributing to increased patient survival [100, 161, 162, 169].

Concerning cancers affecting the male and female genitourinary system, the clinical behavior, evolution, and the types of trials conducted are likely determined by variables similar to those observed in previously mentioned cancers. Additionally, anatomical disposition and hormonal influences on gender-specific organs could impact disease progression and evolution [162], [163], [164, 169, 178].

On the other hand, Melanoma, a type of skin cancer, has been a focus of numerous trials, providing more information on the safety and efficacy of neoantigen-based immunotherapy compared to other cancers described in our study [162, 165, 167, 172, 177, 183].

**Table 8:** Clinical trial which used personalized neoantigen vaccines.

Year	Population	Period of time	Cancer type	Cancer phase	Essay phase
2023 [179]	16 patients	Dec 2019 – Aug 2021	Pancreatic ductal adenocarcinoma		I
2023 [180]	28 patients	Dec 2019 – Aug 2021	Pancreatic ductal adenocarcinoma		I
2023 [170]	6 patients	Oct 2019 – Aug 2020	MSS-colorectal cancer	Advanced	I, II
2022 [163]	24 patients	12 months followed up to 5 years	Urothelial carcinoma	Advanced	II
2022 [161]	16 patients	May 2018 – Apr 2019	Non-squamous non-small cell lung cancer	Advanced	I
2022 [169]	14 patients	32 weeks	Non-small cell lung cancer, MSS-colorectal cancer, gastroesophageal adenocarcinoma and urothelial cancer	Advanced	I
2022 [184]	20 patients	5 years since Jul 2019	Genomic unstable solid tumors		I
2022 [172]	12 patients	Oct 2015 follow-up for 5 years	Melanoma	Advanced	I, II
2022 [173]	28 patients	Feb 2018 – May 2021	Different malignant solid tumors	Advanced	I
2022 [164]	24 patients	May 2017 – May 2022	Relapsed ovarian cancer		I
2021 [100]	12 patients	Nov 2017 – Sep 2019	Lung cancer	Advanced	I, II
2021 [175]	7 patients	33 months	Hepatocellular carcinoma		I, II
2021 [181]	28 patients	May 2015 – Nov 2018	Glioma		I
2020 [168]	4 patients	Mar 2018 – Nov 2019	Gastric cancer	Advanced	I, II
2020 [162]	62 patients	23 weeks	Melanoma, non-small cell lung cancer, or bladder cancer	Advanced	I
2020 [176]	16 patients	6 months	Preventive vaccine for patients with lynch syndrome		I, II
2020 [165]	21 patients	104 weeks	Melanoma	Advanced	I
2020 [183]	12 patients	12 weeks follow-up until 26 weeks	Melanoma		I
2020 [177]	13 patients	2 years	Melanoma		II
2020 [178]	27 patients	May 2014 – Jan 2018	Prostate cancer		II
2020 [171]	29 patients	Nov 2016 – Mar 2019	Diffuse midline glioma	Advanced	I, II
2019 [182]	8 patients	20 weeks	Glioblastoma		I
2019 [174]	22 patients	May 2014 – Aug 2016	Head and neck cancer	Advanced	I, II
2019 [166]	12 patients	Sep 2020 – Sep 2028	Pancreatic adenocarcinoma		I
2018 [167]	42 patients	5 years	Melanoma	Advanced	II

## 7 Discussion

We divided the discussion section into three subsection according to each phase we studied in this review: neoantigen candidates detection, neoantigen prioritization and pipelines/clinical trials.

### 7.1 Neoantigen candidates detection

Neoantigen candidates detection delivers neoantigens taking inputs like RNA-seq, and DNA-seq (see Figure 3). Despite, this process is complex, actually they don’t commonly relies on Transformers or deep learning methods.

The alignment process involves utilizing DNA-seq or RNA-seq data, wherein these samples are mapped to a reference genome to know the specific location of reads. This step stands as a cornerstone in contemporary genomic data analysis. Additionally, in this alignment phase, machine learning methods are not commonly applied due to the inherent nature of the problem, which revolves around identifying similarity regions among sequences of bases. There are proposals which focus on DNA-seq clustering or classification. Moreover, a RNA-seq fasta file typically encompasses approximately 1.7 gigabytes and 5.5 G bases, and it is advisable to include a minimum of 12 samples for a robust RNA experiment [1]. Thus, it is impractical to have this amount of information like input for a deep learning technique.

In variant calling methodologies, several tools described in Section 4.2 and associated algorithms are available. Many of these methods focus on identifying regions of the genome where variants have been called [185]. The most widely used tool does not rely on machine learning methods; however, some proposals involve transforming alignment data into images to facilitate the use of convolutional neural networks. Moreover, the concept of representing alignments as images presents new avenues for utilizing Vision Transformers. Currently, there is a limited number of published works proposing the use of Vision Transformers for variant calling.

Furthermore, the majority of neoantigen detection methods have primarily been employed for the identification of single nucleotide variants (SNV) and small insertions or deletions (indels) [6]. However, it is noteworthy that neoantigens derived from SNVs often exhibit substantial similarity to their normal counterparts. Consequently, only a limited proportion of these putative neoantigens are deemed immunogenic [6]. Moreover, several cancer types are related to alternative splicing events, structural variants and fusion genes [18]. Thus, there is a need for further research that integrates SNPs and structural variants, including fusion genes and alternative splicing events, into variant calling methodologies.

The challenges in neoantigen candidate detection can be summarized as follows: (1) Complex Data: Both RNA-seq and DNA-seq data entail vast amounts of bases, posing difficulties for the application of deep learning techniques. Furthermore, inherent error rates in Next-generation sequencing technologies can adversely affect neoantigen detection. (2) Dependency on Variant Caller Tools: Neoantigen detection heavily relies on variant caller tools. However, the variants identified can vary significantly depending on the alignment tools employed, as well as the sequence accessions and versions [63, 186, 187]. (3) Focus on SNPs: While most variant callers are geared towards single nucleotide polymorphisms (SNPs), numerous cancer diseases are associated with structural variants and fusion genes. Despite this, there remains a scarcity of tools that encompass these events, necessitating further research and methodologies for integrating variant call format (VCF) files from multiple tools. On a positive note, efforts to integrate and establish best practices for variant calling tools in cancer research have been advanced by tools such as the Genome Analysis Toolkit (GATK).

### 7.2 Neoantigen prioritization

Neoantigen prioritization is intricately linked to the task of predicting peptide-MHC binding and pMHC-TCR binding affinity, with the peptide serving as the candidate neoantigen. While the formulation of this problem may seem straightforward, its complexity is undeniable. Consequently, extensive research has been conducted in this area, spanning from classical machine learning approaches to cutting-edge deep learning techniques such as Transformers.

However, these methods have limitations, including their dependence on training datasets that overlook posttranslational modifications (PTMs) like phosphorylation, glycosylation, and deamidation, which influence MHC binding specificity. Additionally, several aspects of pMHC biology are still poorly understood. To improve neoantigen detection accuracy, integration with pMHC-TCR studies is essential.

The baseline methods for this task include netMHCpan4.1 and MHCflurry2.0. A recent comparative study of these tools [117] highlighted the superior performance of MHCflurry. However, it’s worth noting that this comparison did not take into account proposals involving Transformer models. Moreover, recent publications have demonstrated that Transformer-based models, such as ESM-GAT [123], CapsNet-MHC [124], STMHCpan [125], and HLAB [127], exhibit superior performance compared to netMHCpan4.1 and MHCflurry. Nevertheless, there has been no benchmarking comparison conducted on these Transformer models, and as of now, there is no universally acknowledged best tool for pMHC binding prediction.

Another significant challenge in pMHC binding prediction is associated with the MHC class. While a considerable portion of research publications has focused on MHC class I, addressing MHC class II presents a greater challenge. MHC class II peptides tend to be longer and exhibit more variability in length compared to MHC class I peptides. Furthermore, datasets for MHC class II are typically smaller in scale than those available for MHC class I, further complicating the development and validation of predictive models for this class. As a result, overcoming these obstacles and improving prediction accuracy for MHC class II binding remains a pressing area of research in immunoinformatics.

Furthermore, the data within the EIDB continues to expand, furnishing us with an ever-expanding wealth of information crucial for training extensive transformer models. Within this domain, notable examples include ProtTrans [28] and ESM-2 [30], advanced protein language models trained on extensive protein datasets. These models hold promise for transfer learning applications aimed at resolving the challenge of pMHC binding prediction. However, Transformers suffer from instability, where variations in the random seed can lead to significant variance in task performance. Moreover, this instability is exacerbated by optimization difficulties that result in vanishing gradients [188]. Another challenge related to transformers, is the high cost of training huge models, it is less accessible to smaller institution or students with limited resources.

Future directions in pMHC binding prediction may involve leveraging the pHLA3D dataset, which provides 3D structures of alpha/beta chains and peptides of MHC-I proteins. This invaluable resource opens avenues for research in pMHC prediction by incorporating insights from 3D protein structure. Additionally, the emergence of 3D protein structure prediction methods like AlphaFold introduces a new perspective for studying pMHC binding prediction. By integrating information from 3D protein structures, researchers can gain deeper insights into the molecular interactions underlying pMHC binding.

In the case of pMHC-TCR binding affinity prediction, a lot of studies focused only on TCR binding prediction to class I pMHC [133, 135], [136], [137], [138, 140], [141], [142], [143], [144, 189], others studies to class II pMHC [134], and some in both classes [132, 139]. TCR-CD4 binds to pMHC class II, while TCR-CD8 binds to pMHC class I. TCR-CD8 contains information to destroy infected cells along with its potential for recognition and direct elimination, causing many to focus on pMHC class I. However, TCR-CD4 plays a critical role in the initiation and maintenance of an immune response, pMHC class II can incorporate many of the same features as pMHC class I, such as TCR expression, processing, binding and recognition [4]. MHC class II has been shown to play a key role in binding, therefore it should be taken into account [190]. Nevertheless, there are some limitations such as the variation of the input size of the sequences, and the limited training samples. This latter influenced the model, AttnTAP [132] had to reduce the complexity of the model to avoid overfitting. Although there are studies that focus on pMHC class II, which improves binding prediction, it is important to conduct more research.

The database is important to train the models, there are public databases, both for pMHC class I and II, the best known are VDJdb [86], Immune Epitope Database (IEDB) [88] and McPAS-TCR [191]. The recent models are very large and need large training databases, some achieve this by creating their database based on samples from other studies, in some cases they make it public and others do not, making it public facilitates the development of new models that improve the prediction. Furthermore, each author obtains its results based on comparisons with other tools and testing its model with different databases, this makes it difficult to know which proposed model is better. In addition, the databases are constantly being updated, benchmarking is necessary.

In the same way as in pMHC binding prediction, the pHLA3D data set will be used to have new research focuses. The extraction of TCR and pMHC specificity is important. It has been shown, based on experiments, that multi-head self-care extract has better characteristics [136, 137]. Transformers have made many advances in natural language processing and have been shown to perform well in predicting pMHC-TCR binding, however they require a large training database, but still achieve good results. Some choose to use pre-trained, such as transfer learning to improve their results, especially when they have access to limited data or to reduce the computational cost of training.

### 7.3 Pipelines and clinical trials

Despite extensive efforts to develop pipelines and algorithms, less than 5 % of the identified neoantigens have been found to activate the immune system [7, 10], [11], [12], [13]. According to the authors of these pipelines, this limitation may be attributed to the omission of critical data integration, such as DNA-seq, RNA-seq, and Mass Spectrometry (MS) data [14]. Notably, many proposed solutions do not incorporate MS data, despite the increasing availability and its application across various facets of bioinformatics.

Furthermore, outdated tools for predicting peptide-MHC (pMHC) binding (at stage 3.1 in Figure 1b) are commonly employed. Most applications still rely on MHCFlurry [15] and NetMHCpan4.1 [16], although newer, high-performance tools based on transformers are now accessible. Moreover, in the context of stage 3.2 (Figure 1b), authors frequently neglect the prediction of pMHC-TCR binding, though many researchers intend to incorporate this aspect in their future work [17].

Perhaps most crucially, the absence of information related to alternative splicing events, structural DNA variations, and gene fusion mutations represents a significant oversight, despite their strong association with various cancer types [18].

Moreover, we included revision of clinical trial studies in this review. In concluded clinical trials, adoptive cell therapy has demonstrated safety, the ability to elicit an effective immune response against cancer, and an improvement in patient outcomes, including extended disease-free intervals and overall survival, especially when used in combination with checkpoint inhibitors. However, it is worth noting that certain limitations and challenges may have affected the trial outcomes. These include conflicts of interest in the majority of the studies [162, 163, 165, 167, 176], [177], [178, 180, 183], a small sample size in some trials [165, 169, 170, 175, 177, 179], and technical difficulties encountered in a subset of the research. Furthermore, it is essential to note that two trials are still ongoing, and their results are not yet available [166, 184].

Furthermore, it is evident that cancer vaccines utilizing tumor neoantigens offer clinical advantages to cancer patients and hold promise as potential therapies. Hence, dedicating time and resources to their research and development is of utmost importance, with the goal of enhancing current techniques or discovering novel approaches.

Although the focus of immunotherapy is based on the molecular characteristics of tumors, which is the most novel and promising trend for cancer treatment, it is precisely these characteristics that give certain types of cancer peculiarities, making them more or less resistant to different types of treatment, including vaccines based on neoantigens. However, therapeutic strategies that consider both aspects – molecular, based on technological advancements, and clinical, through increasingly extensive, frequent, well-designed therapeutic trials with a larger number of participants – would allow for a comprehensive patient treatment with the aim of achieving better results.

The continuous evolution of vaccine development methods, application techniques, sample acquisition, and the analysis of the molecular profile of tumors, accompanied by timely diagnosis and close monitoring, always prioritizing the human aspect alongside technology, will enable the refinement of these treatments and pave the way for the future in the pursuit of increasingly effective therapies.

### 7.4 Final remarks

In general, the utilization of Transformers in neoantigen detection is exceptionally well-suited for variant calling, pMHC binding prediction, and pMHC-TCR interaction. New proposals continue to evolve, consistently demonstrating strong performance; however, the efficacy of these tools can be compromised due to the insufficient volume of data available in databases. Fortunately, the ongoing expansion of data resources, coupled with advancements in Transformers, paves the way for innovative research in these domains. Furthermore, there are numerous pipelines designed for neoantigen detection. Ensuring the updating of these pipelines to incorporate the benefits of new transformer-based methodologies is of paramount importance. This initiative serves as a driving force behind the development of more robust and current software pipelines. Remarkably, despite the relative immaturity of these technologies, clinical trials have already been conducted, yielding generally positive results and offering promise for potential therapeutic interventions.
